# Association of Depression with Uncontrolled Hypertension in Primary Care Setting: A Cross-Sectional Study in Less-Developed Northwest China

**DOI:** 10.1155/2021/6652228

**Published:** 2021-03-27

**Authors:** Lin Wang, Nanfang Li, Mulalibieke Heizhati, Mei Li, Zhikang Yang, Zhongrong Wang, Reyila Abudereyimu

**Affiliations:** ^1^Xinjiang Medical University, No. 393 Xinyi Road, Urumqi 830001, Xinjiang, China; ^2^Hypertension Center of People's Hospital of Xinjiang Uygur Autonomous Region, Xinjiang Hypertension Institute, National Health Committee Key Laboratory of Hypertension Clinical Research, Urumqi, Xinjiang, China

## Abstract

**Background:**

Hypertensive patients commonly experience comorbid depression, which is closely associated with adverse health outcomes. This study aimed to examine the association between depression and uncontrolled hypertension in primary care setting of Northwest China.

**Methods:**

We used a stratified multistage random sampling method to obtain 1856 hypertensives subjects aged ≥18 years among primary care setting in Xinjiang, Northwest China, between April and October 2019. Depression was evaluated by Hospital Anxiety and Depression Scale (HADS), with a cut-off score ≥8. We related depression to uncontrolled hypertension, using multiple logistic regression, adjusting for minimally sufficient adjustment set of variables retrieved from a literature-based directed acyclic graphs (DAGs) and optimal adjustment set of variables derived from the least absolute shrinkage and selection operator (LASSO) regression.

**Results:**

A total of 1,653 (89.1%) patients had uncontrolled hypertension. The prevalence of depression was 14.5% and 7.4% among patients with uncontrolled and controlled hypertension. Depression was associated with 1.12-fold increased odds of uncontrolled hypertension [odds ratio (OR) 2.12, 95% confidence interval (CI): 1.23–3.65]. The association remained significant even after adjusting for the minimal sufficient adjustment sets and the optimal adjustment set of variables.

**Conclusion:**

Depression is significantly associated with uncontrolled hypertension in primary care setting of northwest China. The integrated management of depression and hypertension in the setting might be warranted.

## 1. Introduction

Hypertension is a major risk factor for cardiovascular disease (CVD), which remains a vital public health problem worldwide, especially in the developing countries including China, since it causes heavy social, familial, and economic dysfunctions [1, 2]. Estimates suggest that hypertension affected 31.1% of adults (1.39 billion) worldwide in 2010 [3]. Among those with hypertension, approximately 75% (1.04 billion) live in the developing countries [3]. China is the world's most populous nation and the largest developing country. The prevalence of hypertension is about 27.9% in adult population in China, whereas control rate is only 15.3% [4], which is even unacceptably low (8.2%) in less developed areas, compared with urban population [5].

For hypertensive patients, the blood pressure (BP) level reaching recommended goals is fundamental to improve health outcomes, minimize their impact, prevent further disability, and reduce health care costs [6–8]. Despite the fact that hypertension is one of the most common chronic conditions managed in primary care [9] and the benefit of reducing BP in hypertensive patients has been demonstrated [10, 11], it is uncontrolled in a significant proportion of patients. Several patient- and physician-specific factors have been implicated in preventing patients from achieving recommended BP goals. Low treatment rate, lack of motivation, and poor adherence to antihypertensive drug are the leading patient-related factors [12, 13]. Those factors are also significantly related to depression [14].

A meta-analysis including 41 studies show that the prevalence of depression among hypertensive patients is 26.8% [15]. In addition, patients with comorbid hypertension and depression have an increased risk of cardiovascular-related morbidity and mortality [16, 17]. However, the association of depression with uncontrolled hypertension has received relatively little attention and there is conflicting data. Aaron et al. [18] conducted a study in US adults, which demonstrated that those with depression have higher rates of hypertension control than patients without depression. This may be related to an increase in utilization of health care resource in primary care and specialty visits among patients with depression, whereas Almas et al. [19] found that patients with a comorbid depression in Pakistan were less likely to have BP control. The reasons behind this discrepancy may be related to the different health care accessibility, health care policy, family economic status of participants, and the different adjusting set of confounders in the study analysis.

The association of depression with uncontrolled hypertension is still debatable, and data are scanty from China, especially from the primary care settings. We hypothesize that depression not only is related to hypertension but is a significant contributor to the uncontrolled hypertension as well. Accordingly, our study aimed to determine the association of depressive disorders with uncontrolled hypertension in primary care setting of China.

## 2. Materials and Methods

### 2.1. Site

The study was conducted at primary health care centers including the stock-raising, agricultural, and urban settings at Emin County, a less developed region in Northwest China, with a total population of over 160,000. We selected Emin County as the study site because both its population size and ethnic composition, and average household income are at the mean of all counties in the province. Additionally, previous survey showed local residents have high prevalence of hypertension, and low control rates [20]. Thus, it is an ideal setting for the study the association of depression with hypertensive status among primary care setting from the less developed region in China.

### 2.2. Study Population

**Figure 1:**
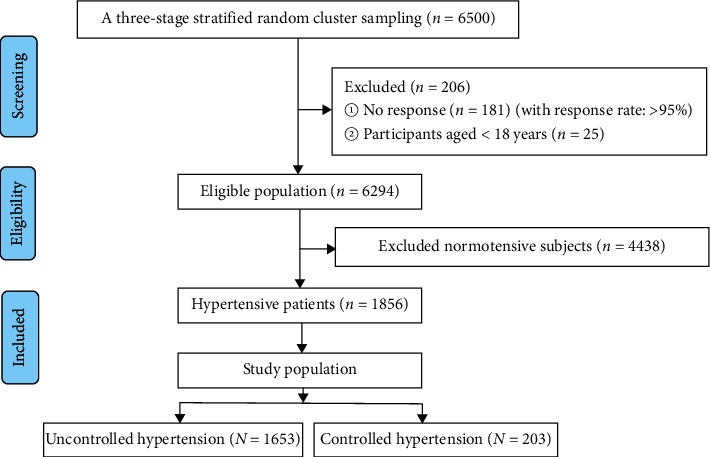
The flow chart of inclusion and screening of surveyed subjects.

This cross-sectional study was conducted between April and October 2019. 40 village and 20 community in four townships and two districts of Emin county were selected using a stratified-cluster sampling method (district/township-community/village-resident), and 6294 subjects aged ≥18 years completed the survey with a response rate of 96.8% (6294/6500). The current analysis included the 1856 hypertensive patients, as in Figure 1.

Subjects were eligible if 1. aged ≥18 years and 2. capable of giving informed consent. Patients with confirmed dementia/Alzheimer's disease and physical disabilities were excluded with the consideration that they could not answer questions. The Ethics Committee of People's Hospital of Xinjiang Uygur Autonomous Region approved the study protocol and consent forms.

### 2.3. Data Collection and Measurement

Trained study staff collected data on demographic characteristics (such as age, sex, and ethnicity), socioeconomic status (occupation, education attainment status, marital status, and personal incomes), behavioral lifestyle factors (cigarette smoking, alcohol intake, physical activity, and sleep quality), and hypertensive history using standardized questionnaires. Other information of comorbidities such as dyslipidemia, diabetes, and cardiovascular disease (including stroke and coronary heart disease) was collected as well.

### 2.4. Blood Pressure (BP) and Anthropometric Variables

Each participant's BP records were measured using with the automatic sphygmomanometer (OMRON HBP-1300, Kyoto, Japan). All participants were measured three times for BP with a 30-second interval between each measurement. The mean value of the three measurements was used for analysis. Height, weight, and waist circumference were also measured by trained staff. Body mass index (BMI) was calculated as weight divided by the square of height (kg/m^2^).

### 2.5. Plasma Glucose and Lipid Measurements

All subjects were fasting for ≥8 h, and a 5 mL fasting blood sample was collected. Then, fasting plasma glucose (FPG), triglycerides (TG), total cholesterol (TC), low-density lipoprotein cholesterol (LDL-C), and high-density lipoprotein cholesterol (HDL-C) were tested at a local primary care center using standard methods.

### 2.6. Hypertension

Hypertension is defined as systolic BP (SBP) ≥140 mmHg, and/or diastolic BP (DBP) ≥90 mmHg, and/or use of antihypertensive agents within two weeks [21]. Treatment was defined as whether they were receiving prescription medication for hypertension during the past two weeks. Control was defined as hypertensive patients who were on antihypertensive treatment and SBP/DBP <140/90 mmHg, otherwise classified as uncontrolled.

### 2.7. Evaluation and Definition of Depression

Depression was evaluated by the Hospital Anxiety and Depression Scale (HADS). The HADS is a 14-item scale and is divided into two subscales directed at either depression (HADS-D) or anxiety (HADS-A). Each subject completed the Chinese version of the HADS questionnaire [22]. Depression was identified as a score of ≥8 in the HADS-D, yielding a sensitivity and specificity of approximately 80% [23]. With depression commonly coexisting with anxiety, anxiety was also evaluated and defined with a cut-off score ≥8 in this analysis, and it was adjusted in the regression model as a covariate.

### 2.8. Definition of Covariates

The participants reported their living region according to the following three categories: agricultural, stock-raising, and urban. Education attainment status was categorized into three categories: primary and lower, junior high, and senior high and higher. Occupation was defined as manual and intelligent. Personal incomes were divided into low incomes ≤¥5000/month (USA$ 700.6/month) and high incomes >¥5000/month, based on Chinese individual income tax rates [24]. Marital status was classified as single, married, or separated. Smoking was coded as yes/no. Alcohol drinking was classified as yes/no. Physical activity was assessed using Chinese version of Global Physical Activity Questionnaire (GPAQ), which was divided into three categories: low, moderate, and high physical activity based on the WHO's (2010) physical activity recommendations [25]. Sleep quality was evaluated by Pittsburgh sleep quality index (PSQI) [26, 27], which was adjusted in the regression model as a continuous variable. BMI was categorized into three groups using the following categories: normal (<24.0 kg/m^2^), overweight (24.0–27.9 kg/m^2^), or obese (≥28.0 kg/m^2^) [28]. Abdominal obesity was defined as WC ≥90 cm in men, and WC ≥85 cm in women. Comorbidity was defined as a combination of two or more diseases: dyslipidemia, diabetes, and CVD.

### 2.9. Statistical Analysis

Descriptive analyses were conducted for all subjects between uncontrolled hypertension and controlled hypertension groups using SPSS 20.0 for Windows (SPSS Inc., Chicago, IL). All continuous variables were summarized as means ± standard deviations (M ± SD), and categorical variables were expressed as frequency (*n*) and proportions (%), and the results were compared using student's *t*-test and the chi-square test to detect the statistical significances, respectively.

Multiple logistic regression models were used to assess association of depression with uncontrolled hypertension adjusted for possible confounders. Odds ratios (OR) were estimated from depression with respect to those without depression together with their CIs (95%) and *P* value. We carried out several logistic regression models based on different adjustment sets.

First, we used directed acyclic graphs (DAGs) to identify suitable minimally sufficient adjustment sets [29]. A minimally sufficient adjustment set consists of the smallest number of variables needed to account for confounding factors [30]. We used the program DAGitty, which is software for drawing and analyzing causal diagrams, to identify the minimally sufficient adjustment set [31]. See the DAGitty user manual for detailed working principles and operation methods [32]. Second, we used the least absolute shrinkage and selection operator (LASSO) regression to select variables which are highly correlated with the outcome and discarded those with negligible effects. LASSO regression was used to screen multidimensional variables; some variables were eliminated because they were not associated with uncontrolled hypertension or because they had strongly collinear with other variables [33]. In addition, we carried out sensitivity analyses in hypertensive patients excluded without treatment. And in the regression model of sensitivity analysis, we further added the duration of hypertension and the number of combinations of antihypertensive drugs to every adjustment set.

## 3. Results

### 3.1. Patient Characteristics

In total, 1856 subjects with mean age 53.7 ± 12.5 years were enrolled with men accounting for 56.7%. As shown in Table 1, most of the enrolled subjects were living in agriculture regions (58.0%) and stock-raising regions (13.0%) and were with primary and lower education status (43.8%). The subjects combined with uncontrolled hypertension were more likely to be youth, men, living in agriculture region, with manual occupation, with marital status as single and married, smoker, and with low PSQI score and low proportion of comorbidity compared with patients with controlled hypertension as *P* value **<**0.05.

### 3.2. The Selection of Adjusted Set

**Figure 2:**
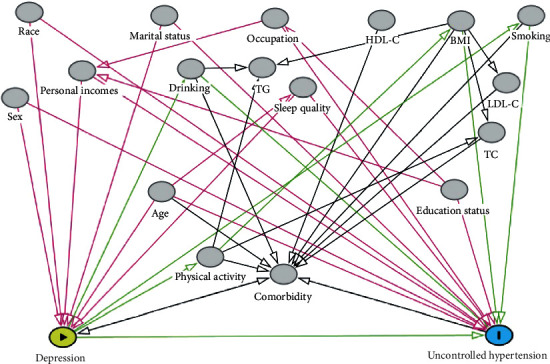
DAG derived from literature and expert knowledge. Nodes represent variables and arrows represent causal associations. Yellow-colored nodes and blue-colored nodes label depression and uncontrolled hypertension, representing exposure and outcome, respectively. Gray-colored nodes represent possible confounding factors. TC, total cholesterol; TG, triglycerides; LDL-C, low-density lipoprotein cholesterol; HDL-C, high-density lipoprotein cholesterol.

Figure 2 shows the results of DAGs describing association between depression and uncontrolled hypertension, based on literature-based report and empirical confirmability. The DAGs algorithm identified the following two minimal sufficient adjustment sets:

1. Age (count), sex, ethnicity, personal incomes, marital status, smoking, alcohol drinking, sleep quality, and BMI
2. Age (count), sex, ethnicity, personal incomes, marital status, smoking, alcohol drinking, physical activity, and sleep quality

**Figure 3:**
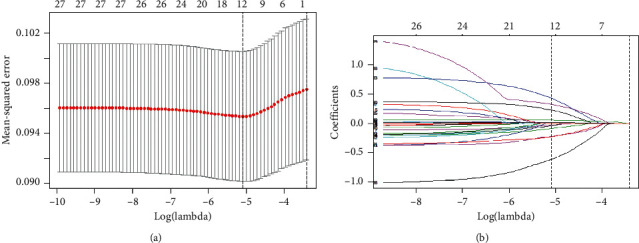
Variable selection using the LASSO binary regression model. Notes: (a) optimal parameter (lambda) selection in the LASSO model used tenfold cross-validation via minimum criteria. The partial likelihood deviance (binomial deviance) curve was plotted versus log(lambda). Dotted vertical lines were drawn at the optimal values by using the minimum criteria and the 1-SE of the minimum criteria (the 1-SE criteria). (b) LASSO coefficient profiles of the 27 features. A coefficient profile plot was produced against the log(lambda) sequence. Vertical line was drawn at the value selected using tenfold cross-validation, where optimal lambda resulted in 12 features with nonzero coefficients. Abbreviations: LASSO, least absolute shrinkage and selection operator; SE, standard error.

We used the LASSO regression model to screen independent related factors of uncontrolled hypertension in all hypertensive subjects. Twelve independent factors were screened out of 27 factors in the study (∼2 : 1 ratio; Figures 3(a) and 3(b)) and were with nonzero coefficients in the LASSO regression model. These factors included age (count), ethnicity, region, marital status, occupation, smoking, heart rate, sleep quality, anxiety, comorbidity, TG, and FPG.

### 3.3. Association of Depression with Uncontrolled Hypertension

On comparison of uncontrolled hypertension and controlled hypertension (Table 2), 239 (14.5%) had depression in uncontrolled hypertension group and 15 (7.4%) in controlled hypertension group with OR (95% CI): 2.12 (1.23–3.65), *P* value = 0.007. In addition, 115 (7.0%) had anxiety in uncontrolled hypertension group and 45 (6.9%) in controlled hypertension group with OR (95% CI) of 1.01 (0.57–1.79), *P* value = 0.974.

On multiple logistic regression for outcome of uncontrolled hypertension and depression as the main exposure variable in model 1, OR (95% CI) for depression was 2.96(1.59–5.48), after adjusting for the first minimal sufficient adjustment sets. In model 2, OR (95% CI) for depression was 2.91(1.57–5.40), after adjusting for the second minimal sufficient adjustment sets. In model 3, OR (95% CI) for depression was 2.88(1.56–5.32), after adjusting for the variable sets derived from LASSO selection. In model 4, OR (95% CI) for depression was 2.83(1.52–5.25), after adjusting for all variables. All *P* values are **<**0.05 (Table 3).

Sensitivity analyses with the same model in hypertensive patients with treatment yielded similar results as in Table 4. Though the OR decreased in the four models, the significance and directionality did not change substantially excepted Model 4.

## 4. Discussion

We report a significant association between depression and uncontrolled hypertension. And the association remained significant even after adjusting for the potential confounders identified by the DAG, LASSO regression, and for all variables. No association could be found between uncontrolled hypertension and anxiety.

The studies that examine the association of depression with uncontrolled hypertension are limited, and the findings are inconclusive and hardly comparable. A case-control study from urban tertiary Hospital Pakistan [19] reported that depression was significantly increased risk of uncontrolled hypertension, which is in line with our study results which report association of depression with uncontrolled hypertension in primary care setting. Nonetheless, in a study conducted in the economically advanced US [18], depression is associated with the increased control rate of hypertension. This contrasts with our study's conclusion. Hypertensive patients with depression show high likelihood of health care utilization, compared with those without depression in US, which may explain, at least in part, the increased control rate of hypertension in this specific patient population [34] and the divergent results among studies. Accordingly, the association of depression with uncontrolled hypertension may be inconsistent in setting with different levels of economic development and medical care.

To our knowledge, this is the first study to evaluate association of uncontrolled hypertension with depression in primary care setting of less developed Northwest China. Considering the potential possibility that confounder bias to the relationship of depression and uncontrolled hypertension is numerous and complex, we collected comprehensive associated variables, and we used DAG and LASSO regression methods, which are the methods used to select precise and appropriate statistical model that control unbiased effect estimates at present. Importantly, the significant association of depression with uncontrolled hypertension still remains after adjusting these confounders derived from DAG and LASSO regression, which may favor the fact that current results have good reliability and stability.

Hypertension has a higher prevalence in Chinese adults, affecting approximately 244.5 million adults, and only 15.3% of these hypertensive patients were controlled [4]. More than 70% of these hypertensive patients are managed in primary health setting in less developed regions of China. Despite the fact that the national health policy has listed hypertension as one of the top priorities of the chronic disease management system [35, 36], the treatment and control rates have been increasing rather slowly in the past 10 years. Current study highlights the association between depression and uncontrolled hypertension. However, depression has been inadequately addressed among hypertensive patients in China, especially in primary health care of less developed rural areas, due to limited knowledge among primary care providers and resources on mental health [37]. Depression accounts for nearly 5.7% of the global burden of disease and it commonly co-exists with chronic disease including hypertension and interacts in complex mechanism to increase the severity and impede treatment, and thus worsens the outcomes of both disorders [38]. Previous studies demonstrate that the management of depression-comorbid medical conditions in primary care settings shows positive health effects in developed countries [39, 40]. However, the effectiveness of integrated care has rarely been studied in developing countries. The current study suggests significant negative relationship between depression and controlled hypertension in primary health care of less developed areas China, but the effect of depression on uncontrolled hypertension is modest. Therefore, it is necessary to clarify the effectiveness and cost efficiency of collaborative management of these two conditions and explore the optimized management algorithm before integrating the management of depression into chronic disease management for hypertension among primary health cares of China.

The current study includes several strengths. Initially, our study contains a wide variety of information on potential confounding factors and strict data collection procedure, and covers wider age range, which may merit the data quality and generalizability. Additionally, DAGs and LASSO regression were used to screen out variable sets needed to be adjusted to form different statistical models to minimize confounding biases in epidemiological studies. This makes our results about the relationship between depression and uncontrolled hypertension more robust and reliable. Finally, our study provides some preliminary evidence that depression may complicate hypertension management, posing a significant clinical and public health challenge in rural China. Further studies on the effectiveness of collaborative management of patients with comorbid hypertension and depression and exploration of the optimized management algorithms are needed.

Inevitably, this study has some limitations. First, due to the cross-sectional nature of the study, we were not able to get a cause-and-effect relationship between depression and uncontrolled hypertension. Previous studies have reported that uncontrolled hypertension might be a risk factor for depression [41]. Further longitudinal study and clinical trial are warranted to determinate the effects of depression and uncontrolled hypertension. Second, the assessment of depression is based on the HADS questionnaire, and not diagnosed by a clinician specializing in psychiatry. Nonetheless, the HADS has been validated in many languages including Chinese version, in countries and settings including general practice and primary care setting [23, 24, 42]. Third, the high number of participants made the gold standard of 24 h measurement of blood pressure not feasible in our study. Nevertheless, the standard method used in this study, with blood pressure which was measured three times by especially trained staff, is better than in most clinical settings. Forth, we failed to collect prescribed treatment for depression and information on adherence to treatment for hypertension. The HADS score and BP level could be influenced by the use of antidepressant medications; and adherence to antihypertensive treatment could affect the status of BP control for hypertensive patients, which may cause information bias in our results.

## 5. Conclusion

Independent associations between depression and uncontrolled hypertension were observed in hypertensive population from developing countries, consistent when adjusting different confounders sets derived from literature-based DAG and LASSO regression models. Health care providers should consider depression when managing hypertensive subjects and find ways to enhance their psychological well-being in order to improve control of hypertension. Further studies will hopefully clarify whether collaborative management would improve control of hypertension in hypertensive patients with depression of the primary care setting in China.

## Figures and Tables

**Table 1:** Characteristics of the subjects according to hypertension status.

	Uncontrolled hypertension (*N* = 1653)	Controlled hypertension (*N* = 203)	Total (*N* = 1856)	*P* value
Age (years)	53.4 ± 12.5	56.8 ± 11.5	53.7 ± 12.5	<0.001
18–44	373 (22.6)	26 (12.8)	399 (21.5)	0.004
45–59	783 (47.4)	102 (50.3)	885 (47.7)	
≥60	497 (30.0)	75 (36.9)	572 (30.8)	

*Sex (n, %)*				
Women	690 (41.7)	113 (55.7)	803 (43.3)	<0.001
Men	963 (58.3)	90 (44.3)	1053 (56.7)	

*Regions (n, %)*				
Agriculture	976 (59.0)	100 (49.3)	1076 (58.0)	0.004
Stock-raising	218 (13.2)	24 (11.8)	242 (13.0)	
Urban	459 (27.8)	79 (38.9)	538 (29.0)	

*Education attainment status (n, %)*				
Primary and lower	719 (43.5)	94 (46.3)	813 (43.8)	0.364
Junior high	604 (36.5)	74 (31.5)	668 (36.0)	
Senior high and higher	330 (20.0)	45 (22.2)	375 (20.2)	

*Occupation (n, %)*				
Manual	1261 (76.3)	142 (70.0)	1403 (75.6)	0.047
Intelligent	392 (23.7)	61 (30.0)	453 (24.4)	

*Ethnicity (n, %)*				
Han	819 (49.5)	113 (55.7)	932 (50.2)	0.173
Kazakh	569 (34.4)	57 (28.1)	626 (33.7)	
Others	265 (16.0)	33 (16.3)	298 (16.1)	

*Personal incomes*				
≤¥5000/month	1586 (95.9)	196 (96.6)	1782 (96.0)	0.678
>¥5000/month	67 (4.1)	7 (3.4)	74 (4.0)	

*Marital status (n, %)*				
Single	76 (4.6)	2 (1.0)	78 (4.2)	<0.001
Married	1346 (81.4)	155 (76.4)	1501 (80.9)	
Separated	231 (14.0)	46 (22.7)	277 (14.9)	

*Smoking (n, %)*				
No	984 (59.5)	143 (70.4)	1127 (60.7)	0.003
Yes	669 (40.5)	60 (29.6)	729 (39.3)	

*Alcohol dinking (n, %)*				
No	1422 (86.0)	281 (89.2)	1603 (86.4)	0.219
Yes	231 (14.0)	22 (10.8)	253 (13.6)	

Heart rate (bpm)	76.50 ± 11.42	75.07 ± 10.91	76.34 ± 11.37	0.092

*Physical activity (n, %)*				
Low	292 (17.7)	35 (17.2)	327 (17.6)	0.995
Median	913 (55.2)	111 (54.7)	1024 (55.2)	
High	448 (27.1)	57 (28.1)	505 (27.2)	

PSQI score	4.71 ± 3.82	6.00 ± 3.54	4.8 ± 3.8	<0.001

Body mass index (kg/m^2^)	27.37 ± 4.48	27.26 ± 4.26	27.4 ± 4.5	0.741

BMI: <23.9 kg/m^2^	366 (22.1)	45 (22.2)	411 (22.1)	0.975
BMI: 24.0–27.9 kg/m^2^	607 (36.7)	76 (37.4)	683 (36.8)	
BMI: ≥28.0 kg/m^2^	680 (41.1)	82 (40.0)	762 (41.1)	

Abdominal circumference (cm)	92.02 ± 11.85	92.18 ± 11.77	92.0 ± 11.8	0.856
*Abdominal obesity (n, %)*				
No	605 (36.6)	69 (34.0)	674 (36.3)	0.466
Yes	1048 (63.4)	134 (66.0)	1182 (63.7)	

*Comorbidity (n, %)*				
No	1500 (90.7)	172 (84.7)	1672 (90.1)	0.007
Yes	153 (9.3)	31 (15.3)	184 (9.9)	

*Blood pressure (mmHg)*				
Systolic blood pressure	154.04 ± 17.77	123.66 ± 10.23	150.7 ± 19.6	<0.001
Diastolic blood pressure	92.75 ± 12.64	77.00 ± 8.80	91.0 ± 13.2	<0.001
FPG (mmol/L)	5.92 ± 2.45	5.71 ± 1.71	5.89 ± 2.38	0.246
TC (mmol/L)	4.99 ± 1.07	5.01 ± 1.08	5.00 ± 1.07	0.825
TG (mmol/L)	1.71 ± 1.40	1.50 ± 0.97	1.68 ± 1.36	0.046
LDL-c (mmol/L)	2.33 ± 0.37	2.32 ± 0.31	2.33 ± 0.36	0.736
HDL-c (mmol/L)	1.56 ± 0.18	1.56 ± 0.18	1.56 ± 0.18	0.709

PSQI, Pittsburgh sleep quality index; FPG, fasting plasma glucose; TC, total cholesterol; TG, triglycerides; LDL-C, low-density lipoprotein cholesterol; HDL-C, high-density lipoprotein cholesterol.

**Table 2:** Association of depression and anxiety in patients with uncontrolled hypertension.

	Uncontrolled hypertension (*N* = 1653)	Controlled hypertension (*N* = 203)	OR (95% CI)	*P* value
Depression	239 (14.5)	15 (7.4)	2.12 (1.23–3.65)	0.007
Nondepression	1414 (85.5)	188 (92.6)		
Anxiety	115 (7.0)	14 (6.9)	1.01 (0.57–1.79)	0.974
Nonanxiety	1538 (93.0)	189 (93.1)		

OR, odds risk; CI, confidence interval.

**Table 3:** Adjusted models for association of depression with uncontrolled hypertension (*N* = 1856).

	Model 1 OR (95% CI)	*P* value	Model 2 OR (95% CI)	*P* value	Model 3 OR (95% CI)	*P* value	Model 4 OR (95% CI)	*P* value
*Depression*								
No	1		1		1		1	
Yes	2.96 (1.59–5.48)	0.001	2.91 (1.57–5.40)	0.001	2.88 (1.56–5.32)	0.001	2.83 (1.52–5.25)	0.001

Model 1: adjusted the first minimal sufficient adjustment set; model 2: adjusted the second minimal sufficient adjustment set; model 3: adjusted for variable set derived from LASSO selection; model 4: adjusted for all variables.

**Table 4:** Adjusted models for association of depression with uncontrolled hypertension excluded “without treatment” (*N* = 712).

	Model 1 OR (95% CI)	*P* value	Model 2 OR (95% CI)	*P* value	Model 3 OR (95% CI)	*P* value	Model 4 OR (95% CI)	*P* value
*Depression*								
No	1		1		1		1	
Yes	2.07 (1.09–3.95)	0.027	1.97 (1.03–3.75)	0.039	1.85 (1.01–3.39)	0.047	1.91 (0.99–3.70)	0.055

Model 1: adjusted the first minimal sufficient adjustment set, plus duration of hypertension and the number of combinations of antihypertensive drugs; model 2: adjusted the second minimal sufficient adjustment set, plus duration of hypertension and the number of combinations of antihypertensive drugs; model 3: adjusted for variable set derived from LASSO selection, plus duration of hypertension and the number of combinations of antihypertensive drugs; model 4: adjusted for all variables, plus duration of hypertension and the number of combinations of antihypertensive drugs.

## Data Availability

Materials included in the manuscript, excluding the relevant raw data, will be made freely available to any researchers who wish to use them for noncommercial purposes, while preserving any necessary confidentiality and anonymity.
